# Identification and expression profile analysis of NUCLEAR FACTOR-Y families in *Physcomitrella patens*

**DOI:** 10.3389/fpls.2015.00642

**Published:** 2015-08-19

**Authors:** Fang Zhang, Min Han, Qiang Lv, Fang Bao, Yikun He

**Affiliations:** College of Life Science, Capital Normal UniversityBeijing, China

**Keywords:** transcription factors, temporal and spatial expression, abiotic stress, gene regulation, abscisic acid

## Abstract

NUCLEAR FACTOR Y transcription factors belong to a multimember family and consist of NF-YA/B/C subunits. Members of the NF-Y family have been reported to regulate physiological processes in plant. In this study, we identified and annotated two NF-YA, nine NF-B, and twelve NF-YC proteins in the genome of *Physcomitrella patens*. Analyses of conserved domains demonstrated that PpNF-YA/B/C shared the same conserved domains with their orthologous proteins in *Arabidopsis, O. sativa* and mouse. Expression profiles indicated that *PpNF-Ys* were widely expressed in different tissues and developmental stages of *P. patens* throughout protonema and gametophores. The majority of *PpNF-Y* genes were responsive to abiotic stress via either ABA-independent or -dependent pathways. Some of ABA-regulated *PpNF-Y* expression were mediated by ABI3. To our knowledge, this study was the first to evaluate NF-Y families in *Physcomitrella patens*, and provides a foundation to dissect the function of PpNF-Ys.

## Introduction

NUCLEAR FACTOR Y (NF-Y) transcription factors (TFs), also known as Heme-associated proteins (HAPs) and CCAAT box binding factors (CBFs), are important regulators of numerous plant developmental processes and stress-induced responses. The NF-Y transcription factor is consists of three subunits: NF-YA (also known as CBF-B/HAP2), NF-YB (CBF-A/HAP3), and NF-YC (CBF-C/HAP5) (Mantovani, 1999). Each subunit of the NF-Y complex is encoded by a gene family in angiosperms (Stephenson et al., 2007; Siefers et al., 2009). For example, the *A. thaliana* genome encodes 36 *NF-Ys* paralogs (Siefers et al., 2009) and *O. sativa* harbors at least 30 *NF-Y* genes (Thirumurugan et al., 2008). At present, the NF-Ys families have been studied in animals and flowering plants (Matuoka and Chen, 2002; Dolfini et al., 2012; Rípodas et al., 2014), they were identified to play important roles in controlling florescence (Kumimoto et al., 2010; Li et al., 2011), nodule formation (Combier et al., 2006), ABA (abscisic acid)/blue light responses (Chen et al., 2007; Nelson et al., 2007; Li et al., 2008; Liu and Howell, 2010; Liang et al., 2014), chloroplast biosynthesis (Miyoshi et al., 2003) and stress tolerance (Warpeha et al., 2007; Yamamoto et al., 2009).

Increasing evidence shows that NF-Y subunits are involved in abiotic stress responses in monocots and dicots. Microarray data showed that accumulation of *TaNF-YA1* reduced statistically in response to drought stress in wheat leaves (Stephenson et al., 2007). The *nf-ya5* mutants are failed to bear drought stress, whereas, overexpression of this gene enhances drought tolerance (Li et al., 2008). In soybean (*G. max*), overexpression of *GmNFYA3* was found to reduce leaf water loss and enhanced drought tolerance (Ni et al., 2013). *AtNFYA1-RNAi* lines were insensitive to salt stress (Li et al., 2013).

There is also research on the NF-YB family. For example, *AtNF-YB2* was found to be up-regulated by osmotic stress (Chen et al., 2007). In barley (*H. vulgar*), *HvNF-YB3* transcript levels were induced by drought and *HvNF-YB1* and *HvNF-YB4* showed a significant decrease in transcription level after application of ABA. *HvNF-YB5*, however, showed a great increase in transcription level in response to high salt stress (Liang et al., 2012). *LEC1* (LEAFY COTYLEDON1, also *NF-YB9)* and *L1L* (LEAFY COTYLEDON1-LIKE, also *NF-YB6*) are involved in *A. thaliana* embryo development and response to ABA (Warpeha et al., 2007). Similar, to *AtNF-YA5* (Li et al., 2008), overexpression of *AtNF-YB1*, a non-LEC1 member of the NF-YB family, increased plant tolerance to drought stress (Nelson et al., 2007). Overexpression of *ZmNF-YB2* not only improved the drought resistance, but also increased production in withholding water conditions (Nelson et al., 2007).

NF-YCs were responsive to abiotic stress. *AtNF-YC2* could be statistically increased in response to many environmental stimuli, such as light, heat, and drought stress (Hackenberg et al., 2012). Another study found that *NF-YC1, -YC5, -YC2*, and *-YC3* greatly increased under dry, salt and ABA treatments in *B. napus* (Liang et al., 2014). Even though there are not any functional studies of these genes so far, it is likely that this gene family should also be associated with drought and/or ABA treatments in angiosperms.

The moss *Physcomitrella patens* is an emerging land plant model with a fully sequenced genome (Rensing et al., 2008). Its unique phylogenetic position, between green algae and angiosperms, enables the study of the evolution of gene function in plants (Rensing et al., 2008) and it has been widely used to study defense mechanisms against abiotic stress (Frank et al., 2005; Cuming et al., 2007; Qudeimat et al., 2008; Komatsu et al., 2013). It has been reported that phytohormone ABA is required in both vascular plants and non-vascular bryophytes to protect vegetative tissue during water stress by activating downstream gene transcription through transcription factors, such as ABSCISICACID INSENSITIVE 3 (ABI3) (Khandelwal et al., 2010). So far, six NF-YB genes had were cloned in *P. patens* (Xie et al., 2008) and the expression of *PpNF-YAs, -YB1-5*, and *-YC1-5* in response to ABA in protonemal tissues was examined (Yotsui et al., 2013). Also, PpNF-YC1 and -YB4 act synergistically with ABI3A to promote transcriptional levels of PpLEA1 with/without ABA treatment (Yotsui et al., 2013). However, the functions of other *NF-Y* genes have not been systematically investigated in *P. patens*, especially in response to abiotic stress, such as salt, osmotic and desiccation stress. Unlike protonema, mature gametophyte has more complicated structure, including rhizoid, buds, stems, and leaves, and is more similar to the structure of higher land plants. Therefore, we selected mature gametophyte tissues as materials to exploit the functions of those *PpNF-Y* genes in response to abiotic stress.

In this study, we identified two *NF-YAs*, nine *NF-YBs* and twelve *NF-YCs* in *P. patens* using sequence information from *A. thaliana*, and analyzed the evolutionary relationship of these genes with their ortholog in *O. sativa* and *A. thaliana*. Subsequently, we systematically determined the expression profile of each NF-Y gene in different developmental stages and under abiotic stress, including NaCl, mannitol, and desiccation treatments. Also, our results suggest that most PpNF-Y members are regulated by abiotic stress in an ABI3-mediated ABA-dependent or ABA-independent manner.

## Materials and methods

### Identification PpNF-Y family members in *P. patens*

Thirty *A. thaliana* NF-Y CDS sequences were retrieved from the *A. thaliana* TAIR database (http://www.arabidopsis.org). The BLASTN program was used with an *E*-value cut-off of 3.8e^−25^ to identify predicted PpNF-Y sequences using *P. patens* database (http://www.phytozome.net/search.php?show=blast&method=Org_Ppatens_v1.6). The primers of each *PpNF-Y* cDNA sequence were designed with Primer Premier 5.0 (Table S1). And the PCR products obtained from cDNA amplification using PrimeSTAR HS DNA polymerase (TaKaRa, R010A, Japan) and followed by sequencing.

### Phylogenetic trees and conservation domains analyses

The phylogenetic trees of KfNF-Y, PpNF-Y, AtNF-Y, and OsHAP were constructed by the Neighbor-Joining method using conserved and full-length amino acid sequences, respectively (MEGA; version 5.05), and the parameters were p-distance model and 1000 bootstrap replicates. Multiple sequence alignments were implemented using the conserved and full-length amino acid sequences, respectively by Clustal_X software. The sequences of KfNF-Ys, AtNF-Ys and OsNF-Ys were gained from NCBI database (http://blast.ncbi.nlm.nih.gov/Blast.cgi), TAIR database (http://www.arabidopsis.org/) and Orygenes_DB (http://orygenesdb.cirad.fr/), respectively.

### Plant material and growth conditions

The Gransder Wood strains of *P. patens* was used as the wild type (Rensing et al., 2008). The triple deletion mutants of the *PpABI3* (*abi3a/b/c*) was described previously (Khandelwal et al., 2010). Cultures were grown in 9 cm Petri dish on BCDAT medium with 0.7% Agar (Sigma-Aldrich, A7002, USA), which corresponds to the minimal medium described by Sakakibara et al. (2003). The culture conditions were as follows: 16 h/8 h light/dark cycles, 80 μmol s^−1^ m^−2^ photosynthetically active radiation, 23°C constant temperature. Tissues from distinct stages were harvested, including protonemata tissue which is compose of chloronema and caulonema cells. Initial cells divided to generate buds, making the transition from protonema to juvenile gametophores (1-week old). Juvenile gametophores is compose of young stem, leafy and rhizoid (2-week old). Mature and late stage gametophores were represented by 3-, 4-, and 8-week old plant, respectively. Sporophytes that produce haploid spores (s) (Arazi, 2012).

### Stress treatment

Gametophores tissues grown for 4 weeks on cellophane-layered BCD medium without AT, then treated with 10 μM ABA (Sigma-Aldrich, A4906, USA), 400 mM NaCl (Sigma-Aldrich, S7653, USA), 600 mM mannitol (Sigma-Aldrich, M9546, USA), and were transferred to an empty Petri dish and desiccated inside a laminar flow hood. Then these tissues were collected in 0/2/4/8/12/24 h(s).

### Total RNA isolation and cDNA synthesis

Total RNA was extracted from various tissues using the RNeasy plant mini Kit (QIAGEN, 74106, USA). RNA samples were treated by RNase-Free DNase I (Thermo, EN0525, USA). 1 μg of total RNA was used for the synthesis of first-strand cDNA using oligo (dT) as a primer and SuperScript III Reverse Transcriptase (Invitrogen, 18080-044, USA) according to the manufacturers' protocols.

### Quantitative PCR

qRT-PCR was performed to examine mRNA levels of *PpNF-Y* genes in different developmental stages and various tissues using the 2^−Δ*ΔCT*^ formula described in Applied Biosystems User Bulletin No. 2. Relative mRNA levels of target genes were normalized to *Tublin* and *Actin5*. Real-time PCR was performed on an Applied CFX96 Touch™ realtime PCR system using the SYBR®Premix ExTaqTM Kit (TaKaRa, RR820L, Japan). Each 20.0 μl qRT-PCR reaction included 10.0 μl SYBR® Premix Ex Taq TM (2 ×), 0.5 μl Forward Primer (10 μM), 0.5 μl Reverse Primer (10 μM), and 0.2 μl cDNA. The reaction was initial denaturation at 95°C for 30 s, followed by 42 cycles of 95°C for 5 s and 60°C for 30 s with a final dissociation stage. The data were the mean value from three biological replicates, and every biological replicate was the average value from three technical repeats. Primers for qRT-PCR reactions are reported in Table S1. As a statistical analysis, Student's *t*-test were used with two-tailed distribution.

## Results

### Identification of *P. patens* NF-Y genes

In order to identify *P. patens* analogs of NF-Y proteins, amino acid sequences of *A. thaliana* NF-Ys (Siefers et al., 2009; Petroni et al., 2012) were used to search against the *P. patens* database (http://www.phytozome.net/search.php?show=blast&method=Org_Ppatens_v1.6). The sequences with high similarity were acquired as homologs. Two tentative consensus (TC) sequences similar to *AtNF-YAs*, nine to *AtNF-YBs*, and twelve to *AtNF-YCs* were obtained and their full length cDNA were cloned and confirmed by sequencing. According to nomenclature of NF-Ys in *A. thaliana, T. aestivum* and *B. distachyon*, the genes were named as follows (Table 1).

**Table 1:** **Annotation of the ***P. patens*** NF-Ys families**.

**NF-YA/B family**			**NF-YC family**		
**Current name**	**Locus name**	**Alias**	**Current name**	**Locus name**	**Alias**
NF-YA1	>Pp1s42_174	Phypa_162379	NF-YC1	>Pp1s51_318	Phypa_124620
NF-YA2	>Pp1s31_299	—	NF-YC2	>Pp1s409_32	Phypa_8911
			NF-YC3	>Pp1s315_9	Phypa_150383
NF-YB1	>Pp1s25_89	Phypa_27666	NF-YC4	>Pp1s158_8	Phypa_19460
NF-YB2	>Pp1s302_35	Phypa_96394	NF-YC5	>Pp1s370_63	Phypa_8789
NF-YB3	>Pp1s83_179	Phypa_53068	NF-YC6	>Pp1s159_32	Phypa_53898
NF-YB4	>Pp1s25_10	Phypa_118490	NF-YC7	>Pp1s112_139	Phypa_134432
NF-YB5	>Pp1s462_7	Phypa_36907	NF-YC8	>Pp1s35_42	Phypa_121230
NF-YB6	>Pp1s148_12	Phypa_53069	NF-YC9	>Pp1s143_36	Phypa_54134
NF-YB7	>Pp1s288_38	Phypa_224453	NF-YC10	>Pp1s329_24	Phypa_97592
NF-YB8	>Pp1s181_25	Phypa_141686	NF-YC11	>Pp1s37_218	Phypa_28539
NF-YB9	>Pp1s217_34	Phypa_91907	NF-YC12	>Pp1s149_89	—

—*, not show in P. patens database*.

### Phylogenetic classification of PpNF-Ys

**Figure 1:**
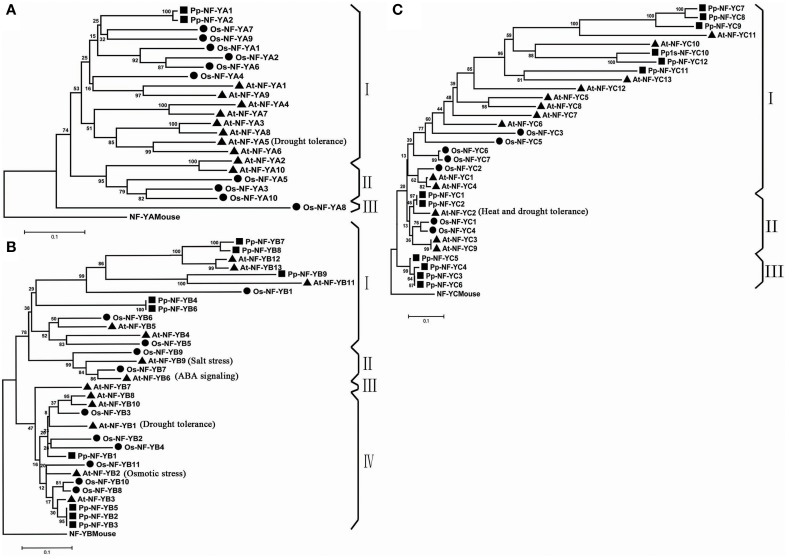
**Phylogenetic tree of NF-YA/B/C subunits**. **(A)** Phylogenetic tree of NF-YA families. **(B)** Phylogenetic tree of NF-YB families. **(C)** Phylogenetic tree of NF-YC families.

The comprehensive phylogenetic analysis was performed to understand the evolutionary status of PpNF-Ys. As showed in the phylogenetic tree (Figure 1A), Pp/Os/AtNF-YAs could be divided into three discrete groups (I–III) comprised of 16, 5, and 1 proteins, respectively. Both two PpNF-YAs subunits were clustered into major group I, and were counterparts with OsNF-YAs. The Pp/Os/AtNF-YB subunits were clustered into four distinct clades (I–IV) consisted of 13, 4, 1, and 15 proteins, respectively (Figure 1B). All PpNF-YBs were clustered into group I or IV, while group II includes AtNF-YB9 (LEAFY COTYLEDON1, AtLEC1), AtNF-YB6 (LEC1-LIKE, L1L), OsNF-YB7, and OsNF-YB9, which agrees with previous work (Siefers et al., 2009; Petroni et al., 2012). Compared to the Pp/Os/AtNF-YB subunits, the Pp/Os/AtNF-YC subunits could be grouped into three clusters (cluster I–III), comprised of 21, 7, and 4 proteins, respectively (Figure 1C). Just as in the PpNF-YA subunits, -YB and -YC also clustered as a closer homolog with *A. thaliana* than OsNF-Y counterparts. The results only minimally change using full-length amino acid sequences (Figures S1A–C).

### Identification of domain conservation

**Figure 2:**
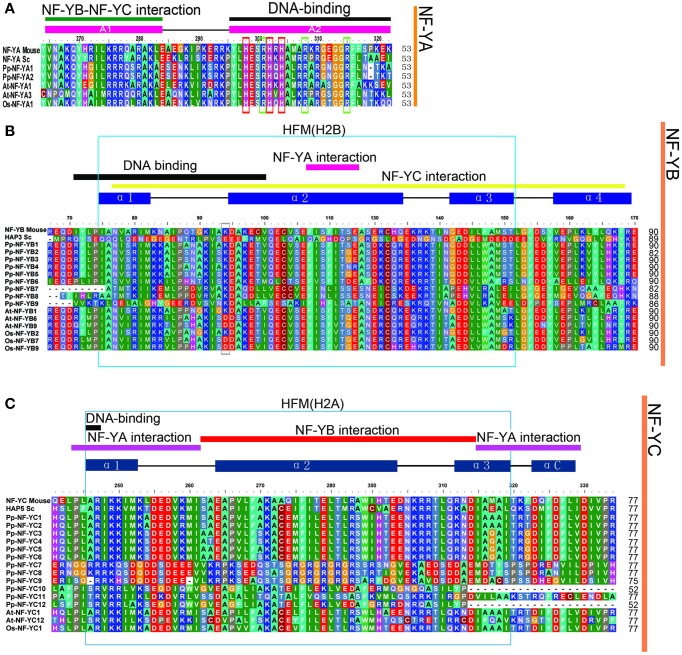
**Multiple sequences alignment of the conversed domains of NF-Ys from ***P. patens***, ***S. cerevisiae, O. sativa***, and ***A. thaliana*****. The functions of referred genes were written in parentheses. **(A)** PpNF-YA family alignment. **(B)** BnNF-YB family alignment. **(C)** PpNF-YC family alignment.

The length of three PpNF-Y subunit families was from 153 aa to 377 aa. Each member of the NF-Y family contains heterodimerization domain and DNA binding domain at the CCAAT site. In order to explore the evolution of the conserved domains in PpNF-Ys, multiple sequence alignment for NF-Ys was applied using full-length protein sequences. The conserved core region of PpNF-YA, -YB, and -YC were ~53, 90, and 77 amino acids in length (Figures 2A–C), respectively, which was similar to Maity and de Crombrugghe (Maity and de Crombrugghe, 1992). The PpNF-YA domain consisted of two subdomains, an A1 domain for the NF-YB and -YC interaction and an A2 domain for DNA binding (Xing et al., 1993, 1994; Mantovani et al., 1994). Three histidine (H) (red box) and an arginine (R) residues (green box) of NF-YA proteins are certainly conserved, and are requisite for DNA binding in mammalian and *S. cerevisiae* (Xing et al., 1993). These residues are also conserved in PpNF-YAs (Figure 2A). PpNF-YB and -YC contained both DNA binding domain and the protein interaction domain (Sinha et al., 1996; Romier et al., 2003). A gray box indicates that the lysine (K) is conserved in non-LEC1-type NF-YB, whereas it changed into an aspartate (D) in a LEC1-type NF-YB. The HFM (histone-fold motif) of the core histone H2B (Figure 2B light blue box) (Mantovani, 1999), which is made up of three alpha-helices and two beta strand-loop domains (solid blue rectangles and black lines were represented on the top of the alignment, respectively) (Arents and Moudrianakis, 1993), was observed (light blue box) in PpNF-YBs. However, it bore slight differences, such as the 66–75 residues in the conserved core in PpNF-YB7, -YB8, and -YB9 (Figure 2B). PpNF-YC were also characterized by a core histone, but were more similar to the HFM of the core histone H2A (light blue box) than H2B (Mantovani, 1999) (Figure 2C).

### Temporal and spatial expression patterns of *PpNF-Y* genes

**Figure 3:**
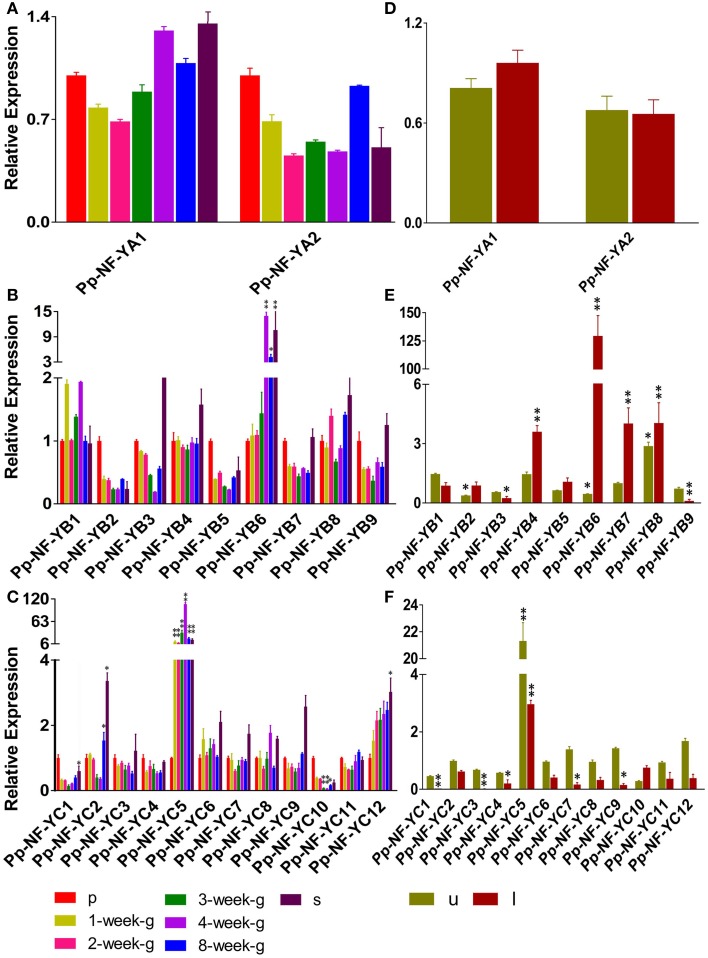
**Temporal and spatial expression patterns of ***PpNF-Ys*** genes**. Total RNA was extracted from protonema (p), from 1-week-/2-week-/3-week-/4-week-/8-week-old gametophores (g), sporophytes (s), and from the upper-part (u), including shoot tip, and lower-part (l), including rhizoids, of 1-week-old gametophores. **(A,D)** Relative transcription abundance of *PpNF-YA*. **(B,E)** Pp*NF-YB*. **(C,F)** Pp*NF-YC*. The expression level of each gene was normalized against that of the protonema. *Tublin* was the housekeeping gene. Error bars indicate SD from three biological replicates. Significant differences were analyzed by *t*-test (^*^P ≤ 0.05 and ^**^P ≤ 0.01).

NF-Y subunit family members are reported to be involved in developmental processes. We examined the developmental- and tissue-specific expression patterns of *PpNF-Y* genes using qRT-PCR. Plants of different developmental stages were harvested: protonema stage, transition stage from protonema to juvenile gametophores (1-week old), juvenile gametophores (2-week old), mature gametophores (3-, 4-, and 8-week old) and sporophytes (s) (Arazi, 2012). The *PpNF-YA*s, *-YB4, -YB8, -YC3, -YC4, -YC6, -YC7, -YC9*, and *-YC11* were constitutively expressed at all developmental stages (Figure 3A, Figure S6A). *PpNF-YB2, -YB5, -YB7, -YB9, -YC1*, and *-YC10* genes were expressed prominently in protonema (Figures 3B,C, Figures S6B,C). Accumulation of *PpNF-YC5* and *-YC12* genes were increased significantly at the transition stage, and was further induced in 2-week old gametophores (Figure 3C, Figure S6C). mRNA level of *PpNF-YB1, -YB6, -YC5*, and *-YC8* expression was highest in adult gametophores (Figures 3B,C, Figures S6B,C) and started to drop in 8-week old gametophore. The expression of *-YC2* was higher in protonema and young gametophores, and lower in adult gametophores. However, its transcriptional level increased dramatically in mature gametophores (Figures 3B,C, Figures S6B,C). Also, the expressions of *PpNF-YB6* and *-YC5* were increased strongly in sporophytes (Figures 3B,C, Figures S6B,C).

The promoter::GUS assay of *AtNF-Ys* showed that each member was detected in a variety of tissues, such as roots, rosette leaves and flowers (Siefers et al., 2009). We wanted to determine whether *PpNF-Ys* exhibited tissue-specific expression patterns as well. We obtained specific tissues of 1-month-old gametophores including the upper-part (u) with the shoot tip, and the lower-part (l) with rhizoids as described previously (Xu et al., 2014a). Accumulation of two *PpNF-YA* genes was not different between the upper and lower part of the gametophore (Figure 3D, Figure S6D). *PpNF-YB2, -YB4, -YB5, -YB6, -YB7*, and *-YB8* were expressed at higher levels in lower-parts of gametophores compared to upper parts, and *-YB6* mRNA level in the lower-part was 125 times higher than that in the upper part. However, *PpNF-YB1, -YB3*, and *-YB9* expressions exhibited the opposite pattern (Figure 3E, Figure S6E). Most of the *PpNF-YCs*, except for *-YC10*, were expressed at lower levels in the lower-part of gametophores compare to that in the upper-part, and *-YC1* and *-YC3* transcriptional levels were too low to be examined in the lower-part (Figure 3F, Figure S6F).

### Expression of *P. patens NF-Y*s transcription in response to different stress

**Figure 4:**
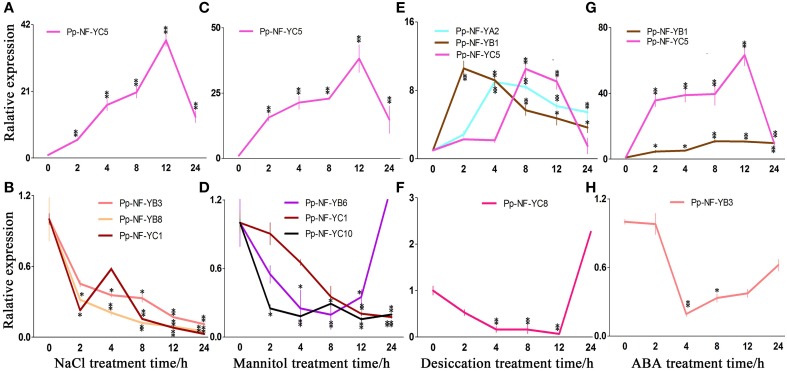
**Typical expression patterns of ***PpNF-Ys*** in wild type plants in response to abiotic stress. (A,B)** *PpNF-Ys* by salt stress. **(C,D)** *PpNF-Ys* by mannitol treatment. **(E,F)** *PpNF-Ys* by desiccation stress. **(G,H)** *PpNF-Ys* by ABA treatment. The expression level of each gene was normalized against that of the untreated control. *Tublin* was the housekeeping gene. Error bars indicate SD from three biological replicates. Significant differences were analyzed by *t*-test (^*^P ≤ 0.05 and ^**^P ≤ 0.01).

Increasing evidence have indicated that *At/TaNF-Y* genes play important roles in response to abiotic stress (Nelson et al., 2007; Stephenson et al., 2007; Li et al., 2008). Accordingly, in order to understand whether *PpNF-Ys* could respond to abiotic stress, we determined the expression of *PpNF-Y* genes under different stress treatments, including salt, desiccation, osmotic stress, and applied exogenous ABA. Only genes showed high level (8 fold and up) of transcriptional alteration before and after treatments were presented in Figure 4, otherwise in the Supplemental Data.

The relative accumulation of *PpNF-YB1, -YB4, -YB6*, and *-YC5* up-regulated after 2 h of NaCl treatment and remained induced up to 12 h of treatment, *-YC5* exhibited a 40-fold of increase after 12 h of treatment (Figure 4A, Figures S2B,C, S7B,C). By contrast, *PpNF-YB3, -YB8*, and *-YC10* reduced significantly with NaCl treatment (Figure 4B, Figures S2B,C, S7B,C). Transcriptional levels of *PpNF-YB2, -YB5, -YC3, -YC4, -YC6*, and *-YC7* were initially inhibited during 2 or 4 h under salt stress, whereas *-YAs, -YC8, -YC9, -YC11*, and *-YC12* up-regulated slightly (Figures S2A–C, S7A–C).

In response to osmotic stress, the transcriptional level of *PpNF-YB1* and *-YB4* was induced to more than 6-fold of the level observed in the untreated control at 12 and 24 h of treatment (Figure 4C, Figures S3B,C, S8B,C). *PpNF-YC5* transcription up-regulated significantly when treated for 12 h and its mRNA level was 48-fold higher compared to the control (Figure 4C, Figures S3C, S8C). Accumulation of *PpNF-YB8, -YC1, -YC2*, and *-YC10* continuously declined, while *-YB6* reduced until 24 h (Figure 4D, Figures S3B,C, S8B,C). Osmotic stress-responsive *PpNF-YB2, -YB3, -YB7, -YB9* -*YC6*, and *-YC11* showed a modest decrease after 8 h of mannitol treatment, while *-YC4, -YC7, -YC8, -YC9*, and *-YC12* increased (Figures S3B,C, S8B,C). The expression of *PpNF-YC3* and *-YAs* remained constant under osmotic stress (Figures S3A,C, S8A,C).

Part of the physiological effects caused by desiccation is due to osmotic stress, but responses to these two kinds of stress are not quite the same. We also identified many *PpNF-Ys* that responded to desiccation. The expression of *PpNF-YB1* and *-YC10* was significantly induced compared to the control after 2 h, while the accumulation of *PpNF-YA2, -YB5, -YC4*, and *-YC6* was induced after 4 h, of *-YB6* and *-YC5* after 8 h and of *-YC7* after 12 h (Figure 4E, Figures S4A–C, S9A–C). The expression of *PpNF-YC8* decreased until 12 h by desiccation (Figure 4F, Figures S4C, S9C). Other desiccation-responsive *PpNF-YA1, -YB3, -YB4, -YC1,-YC2*, and *-YC3* only increased slightly, whereas *-YC11* decreased upon desiccation (Figures S4A–C, S9A–C). *PpNF-YB2, -YB7, -YB8, -YB9*, and *-YC9* transcripts were mostly unaffected by desiccation stress (Figures S4B,C, S9B,C).

### Regulation of NF-Y transcription by ABA

Plants respond to abiotic stress through ABA-dependent and ABA-independent manner. To identify whether *PpNF-Ys* function via the ABA pathway, the expression profiles of all *PpNF-Ys* under ABA treatments were further examined. The relative accumulation of *PpNF-YB1* and *-YC5* was dramatically induced after 2 h of ABA treatment, especially *-YC5*, which exhibited a 60-fold of increase (Figure 4G, Figures S5B,C, S10B,C). In contrast, the accumulation of *PpNF-YB3, -YC1*, and *-YC2* decreased after 2 h and then recovered to untreated levels at 24 h of ABA treatment (Figure 4H, Figures S5B,C, S10B,C). The transcriptional level of *PpNF-YB5, -YB6, -YB8, -YB9, -YC3, -YC4*, -YC6, -YC7, and *-YC8* was induced slightly, but others did not respond to ABA treatment (Figures S5A–C, S10A–C).

### *P. patens NF-Y* members function via the ABI3-dependent pathway

ABI3 is an essential component in the ABA-dependent regulatory network of abiotic stress responses of mosses and angiosperms (Khandelwal et al., 2010; Mönke et al., 2012). *AtNF-YA5* was involved in ABI3-dependent pathways in drought resistance (Li et al., 2008), and *PpNF-YC1* was reported to activate the *PpLEA1* promoter in an ABI3-dependent manner (Yotsui et al., 2013). In order to assess whether there are any other *PpNF-Ys* that act via ABI3 pathway, we compared the expression of *PpNF-Y* genes in wild type and *abi3* loss-of-function mutants (Khandelwal et al., 2010) under abiotic stress.

**Figure 5:**
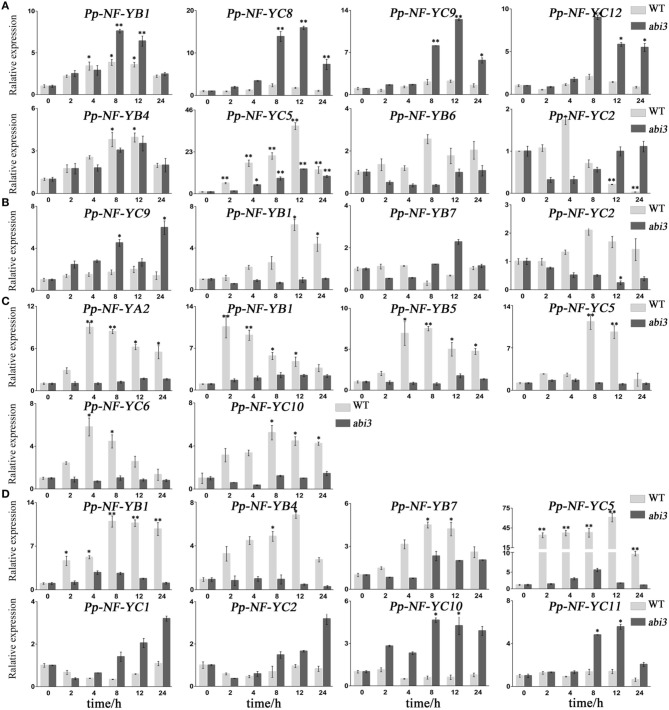
**Typical expression patterns between ***abi3*** mutants and wild type plants in response to abiotic stress**. **(A)** *PpNF-Ys* by salt stress. **(B)** *PpNF-Ys* by mannitol treatment. **(C)** *PpNF-Ys* by desiccation stress. **(D)** *PpNF-Ys* by ABA treatment. The expression level of each gene was normalized against that of the untreated control. *Tublin* was the housekeeping gene. Error bars indicate SD from three biological replicates. Significant differences were analyzed by *t*-test (^*^P ≤ 0.05 and ^**^P ≤ 0.01).

In NaCl treatments, the accumulation of *PpNF-YB4* and *PpNF-YC5* mRNA was lower in *abi3* mutants than that of wild type, while *PpNF-YB1, -YC8, -YC9*, and *-YC12* was higher in *abi3* mutants (Figure 5A, Figures S2E,F, S7E,F). The expression of *PpNF-YB6* and *-YC2* exhibited an opposite pattern (Figure 5A, Figures S2E,F, S7E,F). In response to osmotic stress, transcriptional levels of most *PpNF-Y* genes decreased in *abi3* mutants, with only *-YC9* enhanced after a long time (12 h) of treatment (Figure 5B, Figures S3E,F, S8E,F). The expression of *PpNF-YB1, -YB7*, and *-YC12* had an opposite trend for *abi3* mutants and wild type plants during mannitol treatment (Figure 5B, Figures S3E,F, S8E,F). Many *PpNF-Ys* were induced during the desiccation process (Figure 5C, Figures S4A–C, S9A–C). By contrast, in *abi3* mutants, most *PpNF-Ys* exhibited less alteration of transcriptional levels (Figures S4D–F, S9D–F). For example, *PpNF-YA2, -YB1, -YB5, -YC5, -YC6*, and *-YC10* decreased dramatically in *abi3* mutants under desiccation stress, while *PpNF-YB7, -YB8*, and *-YB9* remained constant in wild type plants (Figure 5C, Figures S4E,F, S9E,F).

To determine whether ABA regulation of *PpNF-Ys* expression is ABI3-dependent, we treated *abi3* mutants with exogenous ABA and compared the mRNA levels of *PpNF-Ys* with that of wild type. Transcriptional levels of *PpNF-YB1, -YB4, -YB7*, and *-YC5* were lower in *abi3* mutants than wild type under ABA treatment (Figure 5D, Figures S5E,F, S10E,F). *PpNF-YC1, -YC2, -YC10*, and *-YC11* had opposite patterns (Figure 5D, Figures S5F, S10F).

## Discussion

### Duplication diversification of *NF-Ys* genes in *P. patens*

Multiple alignments showed that the conserved domains of PpNF-Y family members, with their protein interaction and DNA binding domains (Xing et al., 1994; Sinha et al., 1996; Romier et al., 2003), are highly similar from mammals (such as mouse) to plants (such as angiosperm). Whereas they vary greatly in N- and C-terminal domains of the same NF-Y family (Lee et al., 2003).

The phylogenetic tree indicated that *PpNF-Y* genes formed several monophyletic groups. This implied that *NF-Y* genes expanded in the moss and angiosperm lineages in parallel. *AtLEC1* (*PpNF-YB9*) is a seed-specific gene, is involved in the late stages of embryogenesis (Xie et al., 2008). The fact that the LEC1-type *NF-YB* gene is absent in the moss genome suggests that functional divergence of *LEC1* emerged in seed plants.

Plant Transcriptional factor Database V3.0 (http://planttfdb.cbi.pku.edu.cn/index.php?sp=Ppa) collected 69,450 transcriptional factors from 87 species, demonstrating that there are 2 *NF-YAs*, 16 *NF-YBs*, and 19 *NF-YCs* in *P. patens*, which are different with 23 *NF-Y* members in this study (Table S2). In order to explain the reasons, we collected all *PpNF-Y* gene sequences predicted in PlantTFBD (version 3.0), and found that our cloned NF-Y genes were predicted as transcriptional factors in PlantTFBD, The fact that the different member of *PpNF-Y* genes due to alternative splicing. For example: *Pp1s25_89V6.1* and *Pp1s25_89V6.2 (PpNF-YB1)*; *Pp1s302_35V6.1, Pp1s302_35V6.2, Pp1s302_35V6.3, Pp1s302_35V6.4, Pp1s302_35V6.5* and *Pp1s302_35V6.6 (PpNF-YB2)*; *Pp1s83_179V6.1* and *Pp1s83_179V6.2 (PpNF-YB3); Pp1s159_32V6.1, Pp1s159_32V6.2; Pp1s315_9V6.1, Pp1s315_9V6.2* and *Pp1s315_9V6.3 (PpNF-YC3); Pp1s370_63V6.1, Pp1s370_63V6.2* and *Pp1s370_63V6.3 (PpNF-YC5);* Pp1s159_32V6.2, *Pp1s159_32V6.3*, and *Pp1s159_32V6.4 (PpNF-YC6*). Alternative splicing occurs as a normal phenomenon in eukaryotes and lead to the rapid expansion of genes (Yuan et al., 2009). Also, this view is agreed with the point of new gene functions, which consider that replication of the gene lead to new gene function, subsequently evolution of the genes number in response to environmental stimuli also result in the new gene function (Francino, 2005).

### *PpNF-Y* genes were expressed in various developmental stage and tissues

Previous studies have shown that *BnNF-YA3,-YA13,-YA14* (Liang et al., 2014) and *AtNF-YA2* play roles in root development and in abiotic stress tolerance (Leyva-González et al., 2012). However, we found that accumulation of *PpNF-YAs* was barely changed in different developmental stages and tissues (Figures 3A,D). Based on the phylogenetic trees presents in Figure 1A, PpNF-YAs have less sequence similarity with *B. napus* and *A. thaliana* members, implied that *PpNF-YAs* may have distinct functions that are still unknown. Or due to still being at the primary stage of evolution, the function of *PpNF-YAs* might not be quite established yet. It is reported that *NF-YB* family members in rice, such as *OsNF-YB2, -YB3*, and *-YB4* were ubiquitously expressed (Miyoshi et al., 2003), but their homologs in Physcomitrella, *PpNF-YB2, -YB5, -YB7*, and *-YB9*, accumulated high mRNA levels in protonema (Figure 3B), suggesting the functional divergence of *NF-YB* between mosses and angiosperms. *AtNF-YC3, -YC4*, and *-YC9* express strongly in vascular tissue and regulate flowering time (Siefers et al., 2009; Laloum et al., 2013). *BnNF-YC2* has a relatively high level of expression in the silique stage (Liang et al., 2014). Our data indicate that *PpNF-YB6* plays functions in mature gametophore and sporophytes stage (Figure 3B), *PpNF-YC5* plays roles in the *P. patens*-specific transition stages from protonema to juvenile gametophores, mature gametophore and sporophytes stage (Figure 3C).

Tissue-specific expression patterns indicated that *PpNF-YB2, -YB4, -YB5, -YB6, -YB7*, and *PpNF-YC10* accumulate more than in the lower-parts of mature gametophores (Figures 3E,F). We assumed that these *PpNF-Ys* might play roles in rhizoid, especially for *PpNF-YB6*, which was induced significantly in the lower-part of gametophores. Whereas, *PpNF-YB1, -YB3*, and *-YB9* expressed more in the upper-part of gametophores. Most *PpNF-YCs*, except for *PpNF-YC1* and *-YC3*, showed higher mRNA level in the upper-part of gametophores. Based on the expression patterns, we assumed that *PpNF-YB1, -YB3, -YB9*, and *-YC*s might perform the functions in shoot tips.

### Regulatory network of stress-responsive *NF-Ys* in *P. patens*

Transcription regulation is critical in plant responses to abiotic stress, and transcription factors are important components in these regulatory networks (Saibo et al., 2009). The fact that the transcriptional factor *NF-Ys* could be induced by different abiotic stress suggests a general role in stress responses. There are reported that support this assumption (Leyva-González et al., 2012). According to their expressing patterns during stress treatments, we classified the PpNF-Ys into several groups. *PpNF-YB1* and *-YC5* up-regulated rapidly within 2 h of stress or ABA treatments (Figure 4). *PpNF-Ys* reached transcriptional peaks after 4 or 8 h of stress or ABA treatments, such as *PpNF-YB4, PpNF-YA2, -YB5, -YB6, -YC4, -YC5, -YC6 -YB1*, and *-YB7*. *PpNF-YC5* and *PpNF-YC7* were induced after 12 h of treatment. These genes might play roles at different stages of stress and/or ABA responses. By contrast, some *PpNF-Ys* genes decreased under abiotic stress, including *PpNF-YB8* in mannitol, *PpNF-YC8* in desiccation and *NF-YB3, -YB8, -YC1, YC10* in NaCl. We assumed that these genes act as negative regulators in responses to abiotic stress.

### *PpNF-Y*s function in abiotic stress responses via ABA-dependent and ABA-independent pathways

Two kinds of signaling pathways are reported to respond to plant abiotic stress: ABA-dependent and ABA-independent pathway. Previous studies showed that some *NF-Y* genes could be activated in an ABA-dependent way, including *AtNF-YA5, ZmNF-YB2*, six *BnNF-YAs*, seven *Bn-NF-YBs*, and two *BnNF-YCs* (Nelson et al., 2007; Li et al., 2008; Xu et al., 2014b).

In Physcomitrella and flowering plants, stress-related genes were induced in an ABA-dependent pathway, in which, ABI3 is a key upstream transcriptional factor that induces a series of transcriptional events (Khandelwal et al., 2010; Hauser et al., 2011). It has been reported that transgenic plants overexpressing *AtNF-YA1* greatly up-regulated expression of ABI3, implying that *NF-YA1* is located upstream of ABI3 (Li et al., 2013). And overexpression lines of *AtNF-YA2, -YA7*, and *-YA10* enhance abiotic stress tolerance via altered ABA perception (Leyva-González et al., 2012). The expression of *PpNF-YB4* and *-YB7* increased in NaCl, mannitol, and ABA treatments and it was higher in wild type plants compared with *abi3* mutants. Accumulation of *PpNF-YC5* was dramatically induced by abiotic stress and ABA treatment and it was lower in *abi3* mutants compared with wild type plants, suggesting that these genes are involved in an ABA-dependent pathway and regulated by ABI3 (Figures S2–S5). The transcriptional level of *PpNF-YA2, -YB5*, and *-YB6* was high under desiccation stress and ABA application (Figures S2, S5); we speculated that these genes responded to desiccation in an ABA-dependent manner. *BnNF-YB11* and *-YB14* are strongly induced by abiotic stress via MYB (myeloblastosis oncogene) and MYC (myelocytomatosis oncogene) factors (Xu et al., 2014b). *PpNF-YB1* was induced by ABA treatment, while its expressional level was higher in *abi3* mutants than wild type plants under salt stress (Figures S2, S5); thus we speculated that *PpNF-YB1* might be regulated by other ABA-related pathways, rather than the ABI3-dependent ones. When ABI3 is compromised, the regulatory effects of these pathways are revealed. By contrast, *PpNF-YB1* was responsive to osmotic and desiccation stress through an ABI3-dependent manner. Environmental stress causes plants to over- and under-express several genes to cope with stress (Khraiwesh et al., 2012). PpNF-YB3 was a negative regulatory factor because it reduced in response to NaCl, mannitol and ABA treatments (Figures S2, S5).

It has been suggested that soybean *NF-YA3* involved in ABA-independent pathways (Ni et al., 2013), and NaCl- and mannitol-responsive *BnNF-YB1* and *-YB9* are not affected by ABA treatment either (Xu et al., 2014b). The existence of ABA-independent pathways in mosses has been intensively proved (Cuming et al., 2007). In our study, NaCl-, mannitol-responsive *PpNF-YC8, -YC9* and desiccation-responsive *PpNF-YC1, -YC2, -YC6, -YC10, -YC11* were not response to ABA treatment (Figures S2, S5), implying that these genes respond to abiotic stress via an ABA-independent pathway. Also, previous report has shown that expression of *PpNF-YAs, -YB1-5*, and *-YC1-4* in response to ABA were not significantly changed, however, *PpNF-YC5* was induced strongly between the wild type and *abi3* mutants by northern blotting (Yotsui et al., 2013). These results have some inconsistency with our study, especially, compared with wild type, the expression of *PpNF-YC5* was a dramatically decreased in *abi3* mutants (Figure 5D). The reason might lie in the materials selected, the protonamal tissues were used in Yotsui's paper, but the gametophores was selected in our study. Based on temporal and spatial expression pattern of *PpNF-Ys* presented in Figure 3 and Figure S6, expressions of those *PpNF-Y* genes were different in various developmental process, implied that they exhibited different degree expression pattern in response to same treatment in various developmental stages.

In conclusion, our work confirmed the existence of PpNF-Y genes in the Physcomitrella genome, and analyzed the transcription pattern of PpNF-Y family members. The present study offers a useful foundation for further studies of PpNF-Y proteins.

### Conflict of interest statement

The authors declare that the research was conducted in the absence of any commercial or financial relationships that could be construed as a potential conflict of interest.
