# Supplementary material for: Identification and expression profile analysis of NUCLEAR FACTOR-Y families in Physcomitrella patens
Source: Front Plant Sci. 2015 Aug 19;6:642. doi: 10.3389/fpls.2015.00642 (PMC4541308; doi:10.3389/fpls.2015.00642)
Supplement: Supplementary file 1 [file DataSheet1.PDF]

Supporting Information

Table S1 Primer used in this study

Name	Sequence for PCR (5'-3')	Sequence for real-time PCR (5'-3')
NF-YA1	ATGCACATCAGCTTTGTCGAC	ATCTTCATCCGCCACAATCC
	TCAGGTGGCCACTGCAGTAT	TCCGACCCACTAACCACTAA
NF-YA2	ATGCAGGCGCCCTCGTC	AGGTATTGTTGCCGCCTATG
	CAGGTGGCAACTGCAGTATGTTC	AGGCTCTTCCTCCATCATCT
NF-YB1	ATGGCTGAGGCTGGAAGTCCTG	GACGATTTACTGTGGGCTATGA
	CTAATAATGTTGAACCTGGTAGTTGC	CAAAGCACCCTCAATCTCTCT
NF-YB2	ATGGCCGATAGCTACGGTCACAAC	TAAGTGCCAGAGGGAGAAGA
	CTACACTCCACGAGGCTGGTTTCC	CTTCAGAGGCTCCACGTAATC
NF-YB3	ATGGCCGACAGTTACGGCCACAAC	GAGTGCGTATCCGAGTTCATC
	TTACACTCCGCGGGGTTGGTTTCC	CCGTTGATCGTCTTCCTCTTT
NF-YB4	ATGGCGGAGGCTGGAAGTCCTGGTA	AGGATCGTTTCCTGCCAATC
	CTAATAATGCTGTACCTGGTAATTGCCAGACTG	GAACTGTCTCCTTCGCATCTT
NF-YB5	ATGGCTGACAGCTACGGCCACAAC	CTGGTGAAGCGTCGGATAAA
	TTACAACCCACGAGACTGATTACTGCCAGATTG	TGTACTCATAGCCCACAACAAG
NF-YB6	ATGGCCGACAGCGGCAGCGACAAA	CGCTGAGCTCCTTGAGATTTA
	TTAGTATGGCGGCCTCATGTGATGCAA	TTGCTAAGATTGCTTTCCATTCC
NF-YB7	ATGGATACTGCCGGCAGTCG	ATCCTGGGTTTCGGAGAGTA
	CTAGTCGGAGTCATTGGCTTGTG	CTCCTTCCTTTGCCCATCTT
NF-YB8	ATGGATACTGCTGCCGGCC	GTCCTGAGAGCACTTGAGATTC
	CTAGCTGGAGTCATTGGCCTGTG	GGGATTCCAGGGTTTCATTCT
NF-YB9	TTAGTTGTCTGTTGTGTCATCATCG	CGAAAGGACCAAGAAGCTAAGA
	ATGGGGGGAGGAGGGG	CAGCAGGGTTATCGTCAACA
NF-YC1	ATGGAGAATCATCAACAGCTT	GGATACACACCGAAGAGAACAA
	ACTATTTGGCGGCAT	GGACGATATCCACCAAGAAGTC
NF-YC2	ATGGAGAACCGTTCACAACTTGC	GCTTAGTTACCAGCACCTTCA
	TTAACTGCTTGGCGGCATACTA	GTCGTTCGCCTGTTCAATTTC
NF-YC3	ATGGCCATCCAGCATCCCC	GCTATCACTAGAGGCGACATTT
	TTACTGCATCTGCTGCTGCTGTT	AACTCCAGACCATGGAACAC
NF-YC4	ATGGCCACGCAGCACGC	GAGACGGACACTACAGAGAAAC
	TTAATACAGTTGCTGCTGTGGTTGC	AGACCATGGTACACCCAAATC
NF-YC5	ATGCGGGCACTCGCGG	GCGTCTCTTTCCTCTGCTAAA
	TCAAAATTTTACTTTTGCAACAAAACA	TTCATCTGTCCTTGCCCTTG
NF-YC6	ATGGCCGCCCAGCAGC	CGCAGCTTCATTATCAGCAAAT
	TCACTGTTGCTGCTGCTCGG	GTGGTAGCTGATGGGTCTTAAA
NF-YC7	ATGAAGAAGAAACTGGACACACG	AGATTCTGAAGATGCTGAGATGG
	TCAAAAGATCAGGGGTGCC	CCGATTTCGGCCTAGGTTTAT
NF-YC8	ATGAAGAAGAAGCTGGACACACG	CTGACGATGCTGAGATGGATAC
	CTAACCATCGTCTTCACAATCATAGT	CCGGTTTGGGTATCGCTTTA
NF-YC9	ATGCCCAGTAATCAATGCCAG	ACCGGTGAGGAAAGAATTAGTG
	TCAGTCATAATCGTCCTCATCGG	GACCTCTACCTGACGTTGATTG
NF-YC10	ATGAGCACCACAAACTCGGAGT	GGCAGGCATCGATCCTTTAT

	CTACTTGTCGGTCATTGAAGCGTT	GGAATCCTCACAGGCACTATATC
NF-YC11	ATGGCTAGGAAGGAGGAGATC	CGTCTCTTGCGCGATTATGT
	TCACGTTTTGAAAAAGATTGTG	GGAATCACCGGAGAAGCATTAG
NF-YC12	ATGAGCACCACTAATTCAGAGTCCG	CGGCAGGCATCAATTCTTTATC
	CTACATGTTCATGCGACGACGAT	GGACAGTGAAGAGTAGGAAACC
Tublin		GAGTTCACGGAAGCGGAGAG
		TCCTCCAGATCCTCCTCATA
Actin5		ACCGAGTCCAACATTCTACC
		GTCCACATTAGATTCTCGCA

Table S2 Annotation of *PpNF-Y* genes in PlantTFBD

Name	Description of Plant TFBD				
PpNF-YA1	Pp1s42_174V6.1	NF-YA	AT1G30500.2	5e-43	nuclear factor Y, subunit A7
PpNF-YA2	Pp1s31_299V6.1	NF-YA	AT1G30500.2	9e-43	nuclear factor Y, subunit A7
PpNF-YB1	Pp1s25_89V6.1	NF-YB	AT4G14540.1	1e-68	nuclear factor Y, subunit B3
	Pp1s25_89V6.2	nuclear factor Y, subunit B3			
PpNF-YB2	Pp1s302_35V6.6	NF-YB	AT4G14540.1	8e-75	nuclear factor Y, subunit B3
	Pp1s302_35V6.2	nuclear factor Y, subunit B3			
	Pp1s302_35V6.3	nuclear factor Y, subunit B3			
	Pp1s302_35V6.4	nuclear factor Y, subunit B3			
	Pp1s302_35V6.5	nuclear factor Y, subunit B3			
	Pp1s302_35V6.6	nuclear factor Y, subunit B3			
PpNF-YB3	Pp1s83_179V6.1	NF-YB	AT4G14540.1	6e-74	nuclear factor Y, subunit B3
	Pp1s83_179V6.2	nuclear factor Y, subunit B3			
PpNF-YB4	Pp1s25_10V6.1	NF-YB	AT4G14540.1	2e-64	nuclear factor Y, subunit B3
PpNF-YB5	Pp1s462_7V6.1	NF-YB	AT4G14540.1	7e-72	nuclear factor Y, subunit B3
PpNF-YB6	Pp1s148_126V6.1	NF-YB	AT4G14540.1	4e-43	nuclear factor Y, subunit B3
PpNF-YB7	Pp1s288_38V6.1	NF-YB	AT5G23090.2	1e-73	nuclear factor Y, subunit B13
PpNF-YB8	Pp1s181_25V6.1	NF-YB	AT5G23090.2	1e-67	nuclear factor Y, subunit B13
PpNF-YB9	Pp1s217_34V6.1	NF-YB	AT2G27470.1	9e-32	nuclear factor Y, subunit B11
PpNF-YC1	Pp1s51_318V6.1	NF-YC	AT1G56170.2	2e-75	nuclear factor Y, subunit C2
PpNF-YC2	Pp1s409_32V6.1	NF-YC	AT1G56170.2	2e-75	nuclear factor Y, subunit C2
PpNF-YC3	Pp1s315_9V6.3	NF-YC	AT3G48590.1	5e-70	nuclear factor Y, subunit C1
	Pp1s315_9V6.2	nuclear factor Y, subunit C1			
	Pp1s315_9V6.3	nuclear factor Y, subunit C1			
PpNF-YC4	Pp1s158_8V6.1	NF-YC	AT3G48590.1	2e-66	nuclear factor Y, subunit C1
PpNF-YC5	Pp1s370_63V6.3	NF-YC	AT1G56170.2	1e-59	nuclear factor Y, subunit C2
	Pp1s370_63V6.2	nuclear factor Y, subunit C4			
	Pp1s370_63V6.3	nuclear factor Y, subunit C2			
PpNF-YC6	Pp1s159_32V6.4	NF-YC	AT3G48590.1	3e-67	nuclear factor Y, subunit C1
	Pp1s159_32V6.2	nuclear factor Y, subunit C1			
	Pp1s159_32V6.3	nuclear factor Y, subunit C1			
	Pp1s159_32V6.4	nuclear factor Y, subunit C1			
PpNF-YC7	Pp1s112_139V6.1	NF-YC	AT3G12480.1	5e-51	nuclear factor Y, subunit C11

PpNF-YC8	Pp1s35_42V6.1	NF-YC	AT5G19490.1	5e-55	Histone superfamily protein
PpNF-YC9	Pp1s143_36V6.1	NF-YC	AT3G12480.1	1e-41	nuclear factor Y, subunit C11
PpNF-YC10	Pp1s329_24V6.1	NF-YC	AT1G07980.1	3e-19	nuclear factor Y, subunit C10
PpNF-YC11	Pp1s37_218V6.1	NF-YC	AT5G43250.1	1e-11	nuclear factor Y, subunit C13
PpNF-YC12	Pp1s149_89V6.1	NF-YC	AT1G07980.1	2e-14	nuclear factor Y, subunit C10

Figure S1. Phylogenetic tree of NF-YA/B/C subunits using full-length amino acid sequences. (A) Phylogenetic tree of NF-YA families. **(B)** Phylogenetic tree of NF-YB families. **(C)** Phylogenetic tree of NF-YC families.

Figure S2. Real-time PCR analysis of *PpNF-YA/B/C* expression in gametophores of wild type and *abi3* in response to salt stress. (A),(B),(C) Real-time PCR analysis of *PpNF-YAs*, *-YBs* and *-YCs* expression in wild type, respectively. **(D),(E),(F)** Real-time PCR analysis of *PpNF-YAs*, *-YBs* and *-YCs* expression in *abi3a/b/c* mutants, respectively. The expression level of each gene was normalized against that of the untreated control. *Tublin* was the housekeeping gene. Error bars indicate SD from three biological replicates.

Figure S3. Real-time PCR analysis of *PpNF-YA/B/C* expression in gametophores of wild type and *abi3* in response to osmotic stress. (A),(B),(C) Real-time PCR analysis of *PpNF-YAs*, *-YBs* and *-YCs* expression in wild type, respectively. **(D),(E),(F)** Real-time PCR analysis of *PpNF-YAs*, *-YBs* and *-YCs* expression in *abi3a/b/c* mutants, respectively. The expression level of each gene was normalized against that of the untreated control. *Tublin* was the housekeeping gene. Error bars indicate SD from three biological replicates.

Figure S4. Real-time PCR analysis of *PpNF-YA/B/C* expression in gametophores of wild type and *abi3* in response to desiccation stress. (A),(B),(C) Real-time PCR analysis of *PpNF-YAs*, *-YBs* and *-YCs* expression in wild type, respectively. **(D),(E),(F)** Real-time PCR analysis of *PpNF-YAs*, *-YBs* and *-YCs* expression in *abi3a/b/c* mutants, respectively. The expression level of each gene was normalized against that of the untreated control. *Tublin* was the housekeeping gene. Error bars indicate SD from three biological replicates.

Figure S5. Real-time PCR analysis of *PpNF-YA/B/C* expression in gametophores of wild type and *abi3* in response to ABA. (A),(B),(C) Real-time PCR analysis of *PpNF-YAs*, *-YBs* and *-YCs* expression in wild type, respectively. **(D),(E),(F)** Real-time PCR analysis of *PpNF-YAs*, *-YBs* and *-YCs* expression in *abi3a/b/c* mutants, respectively. The expression level of each gene was normalized against that of the untreated control. *Tublin* was the housekeeping gene. Error bars indicate SD from three biological replicates.

Figure S6. Temporal and spatial expression patterns of *PpNF-Ys* genes. Total RNA

was extracted from protonema (p), from 1-week-/2-week-/3-week-/4-week-/8-week-old gametophores (g), sporophytes (s) and from the upper-part (u), including shoot tip, and lower-part (l), including rhizoids, of 1-week-old gametophores. **(A),(D)** Relative transcription abundance of *PpNF-YA*. **(B),(E)** Pp*NF-YB*. **(C),(F)** Pp*NF-YC*. The expression level of each gene was normalized against that of the protonema. *Actin5* was the housekeeping gene. Error bars indicate SD from three biological replicates. Significant differences were analyzed by *t* test (*P⩽0.05 and **P⩽0.01).

Figure S7. Real-time PCR analysis of *PpNF-YA/B/C* expression in gametophores of wild type and *abi3* in response to salt stress. (A),(B),(C) Real-time PCR analysis of *PpNF-YAs*, *-YBs* and *-YCs* expression in wild type, respectively. **(D),(E),(F)** Real-time PCR analysis of *PpNF-YAs*, *-YBs* and *-YCs* expression in *abi3a/b/c* mutants, respectively. The expression level of each gene was normalized against that of the untreated control. *Actin5* was the housekeeping gene. Error bars indicate SD from three biological replicates.

Figure S8. Real-time PCR analysis of *PpNF-YA/B/C* expression in gametophores of wild type and *abi3* in response to osmotic stress. (A),(B),(C) Real-time PCR analysis of *PpNF-YAs*, *-YBs* and *-YCs* expression in wild type, respectively. **(D),(E),(F)** Real-time PCR analysis of *PpNF-YAs*, *-YBs* and *-YCs* expression in *abi3a/b/c* mutants, respectively. The expression level of each gene was normalized against that of the untreated control. *Actin5* was the housekeeping gene. Error bars indicate SD from three biological replicates.

Figure S9. Real-time PCR analysis of *PpNF-YA/B/C* expression in gametophores of wild type and *abi3* in response to desiccation stress. (A),(B),(C) Real-time PCR analysis of *PpNF-YAs*, *-YBs* and *-YCs* expression in wild type, respectively. **(D),(E),(F)** Real-time PCR analysis of *PpNF-YAs*, *-YBs* and *-YCs* expression in *abi3a/b/c* mutants, respectively. The expression level of each gene was normalized against that of the untreated control. *Actin5* was the housekeeping gene. Error bars indicate SD from three biological replicates.

Figure S10. Real-time PCR analysis of *PpNF-YA/B/C* expression in gametophores of wild type and *abi3* in response to ABA. (A),(B),(C) Real-time PCR analysis of *PpNF-YAs*, *-YBs* and *-YCs* expression in wild type, respectively. **(D),(E),(F)** Real-time PCR analysis of *PpNF-YAs*, *-YBs* and *-YCs* expression in *abi3a/b/c* mutants, respectively. The expression level of each gene was normalized against that of the untreated control. *Actin5* was the housekeeping gene. Error bars indicate SD from three biological replicates.
