# Supplementary figures and images for: Identification and expression profile analysis of NUCLEAR FACTOR-Y families in Physcomitrella patens
Source: Front Plant Sci. 2015 Aug 19;6:642. doi: 10.3389/fpls.2015.00642 (PMC4541308; doi:10.3389/fpls.2015.00642)

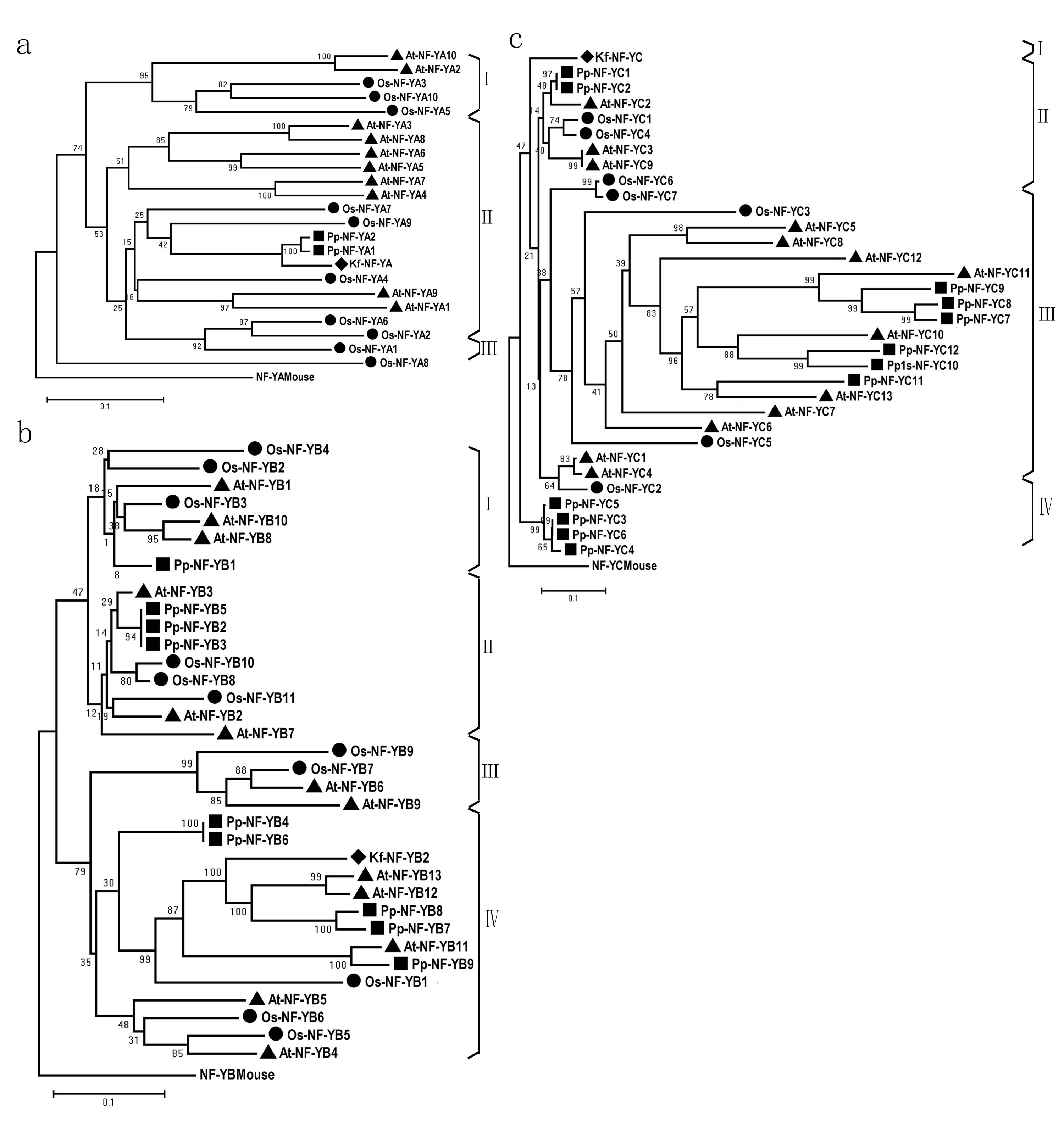

Supplement: Supplementary file 2 [file Image1.JPEG]

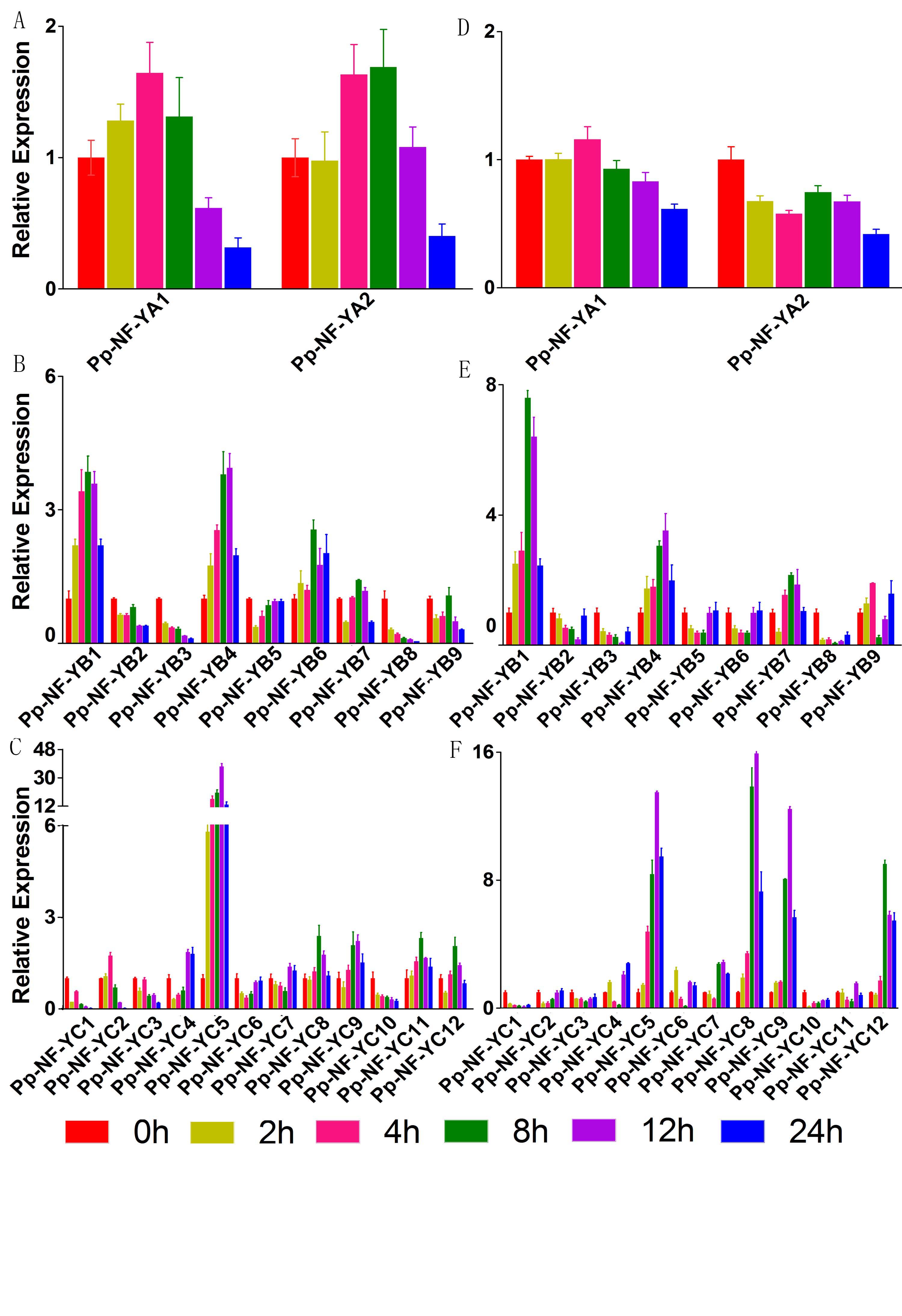

Supplement: Supplementary file 3 [file Image2.JPEG]

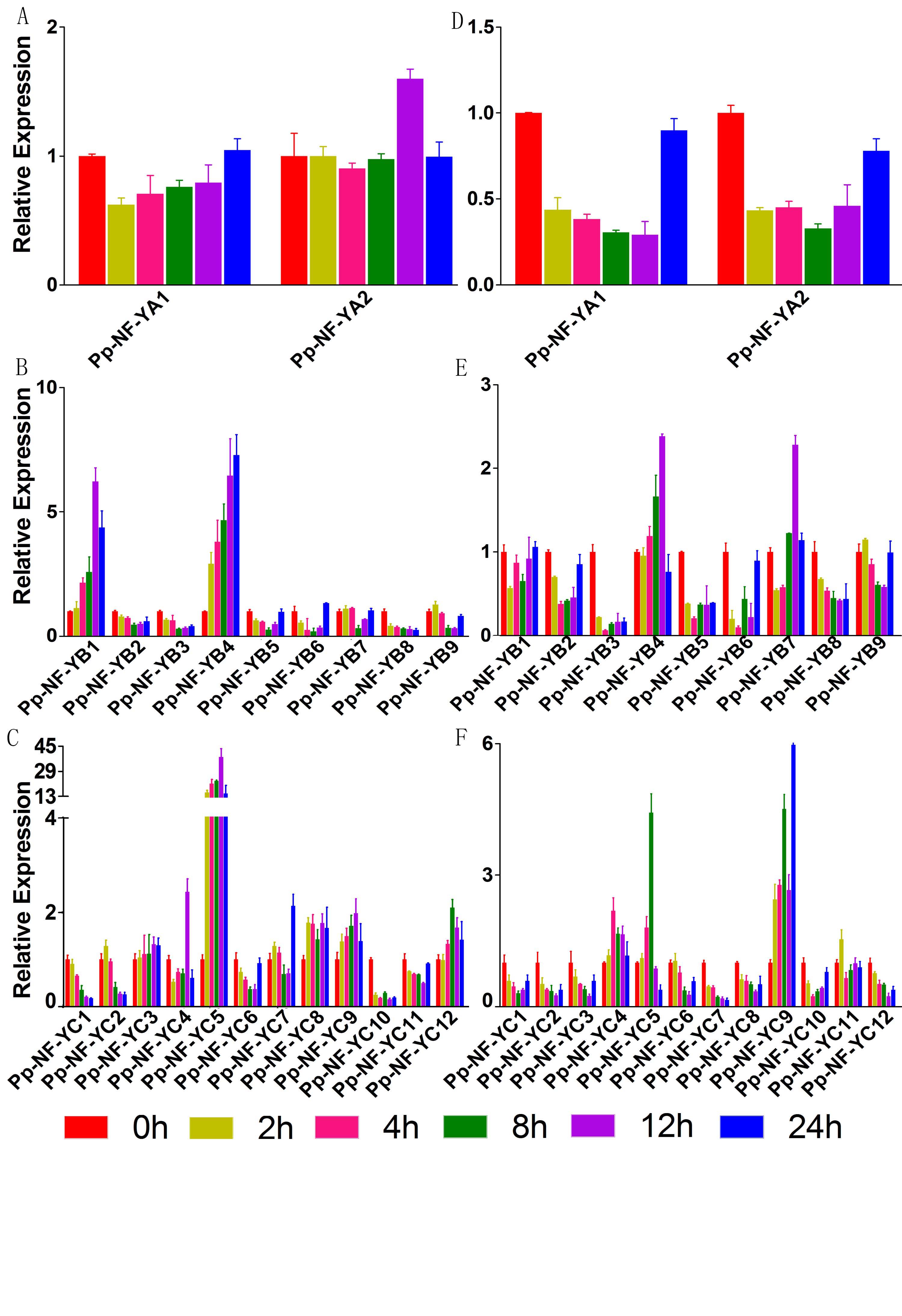

Supplement: Supplementary file 4 [file Image3.JPEG]

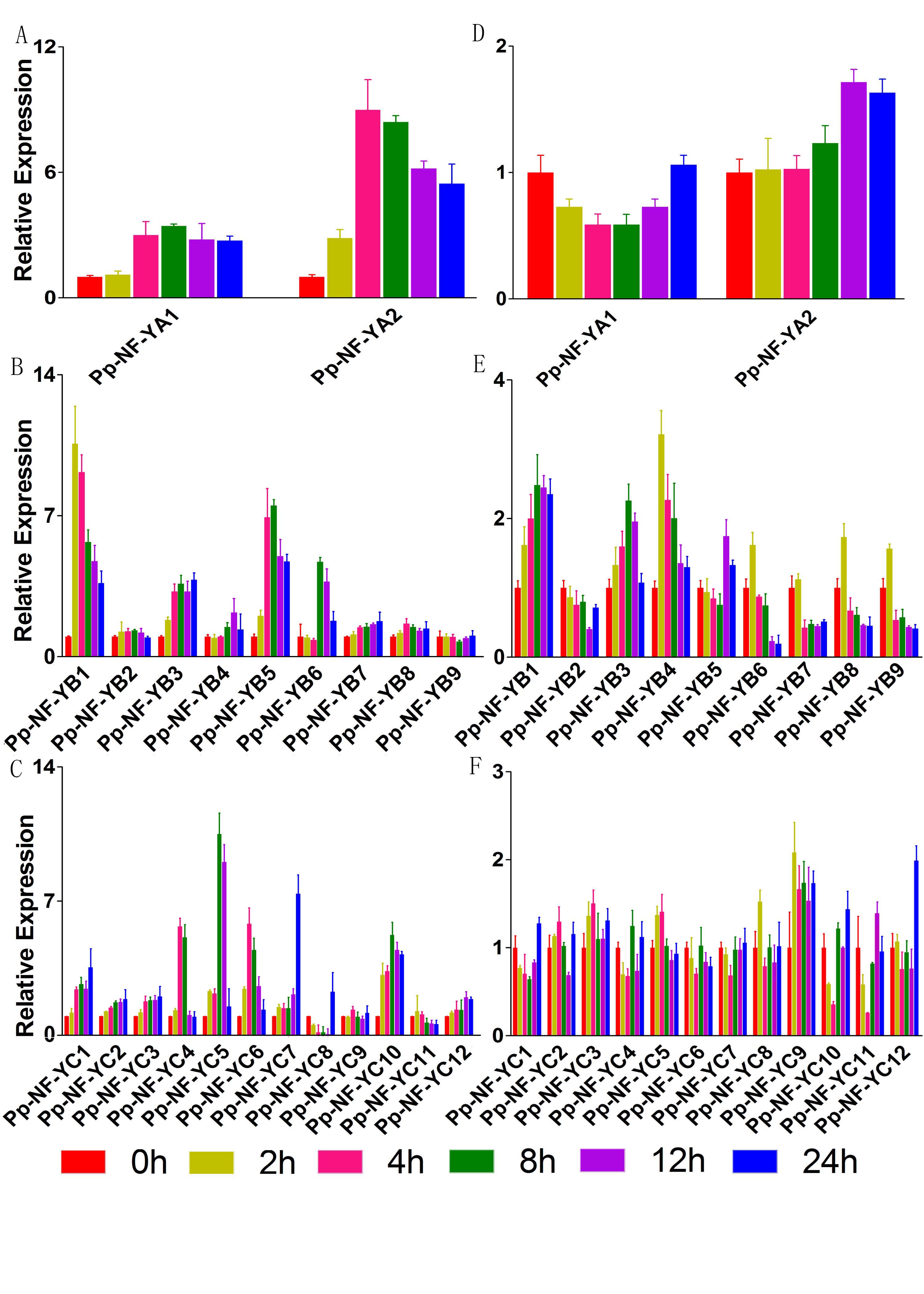

Supplement: Supplementary file 5 [file Image4.JPEG]

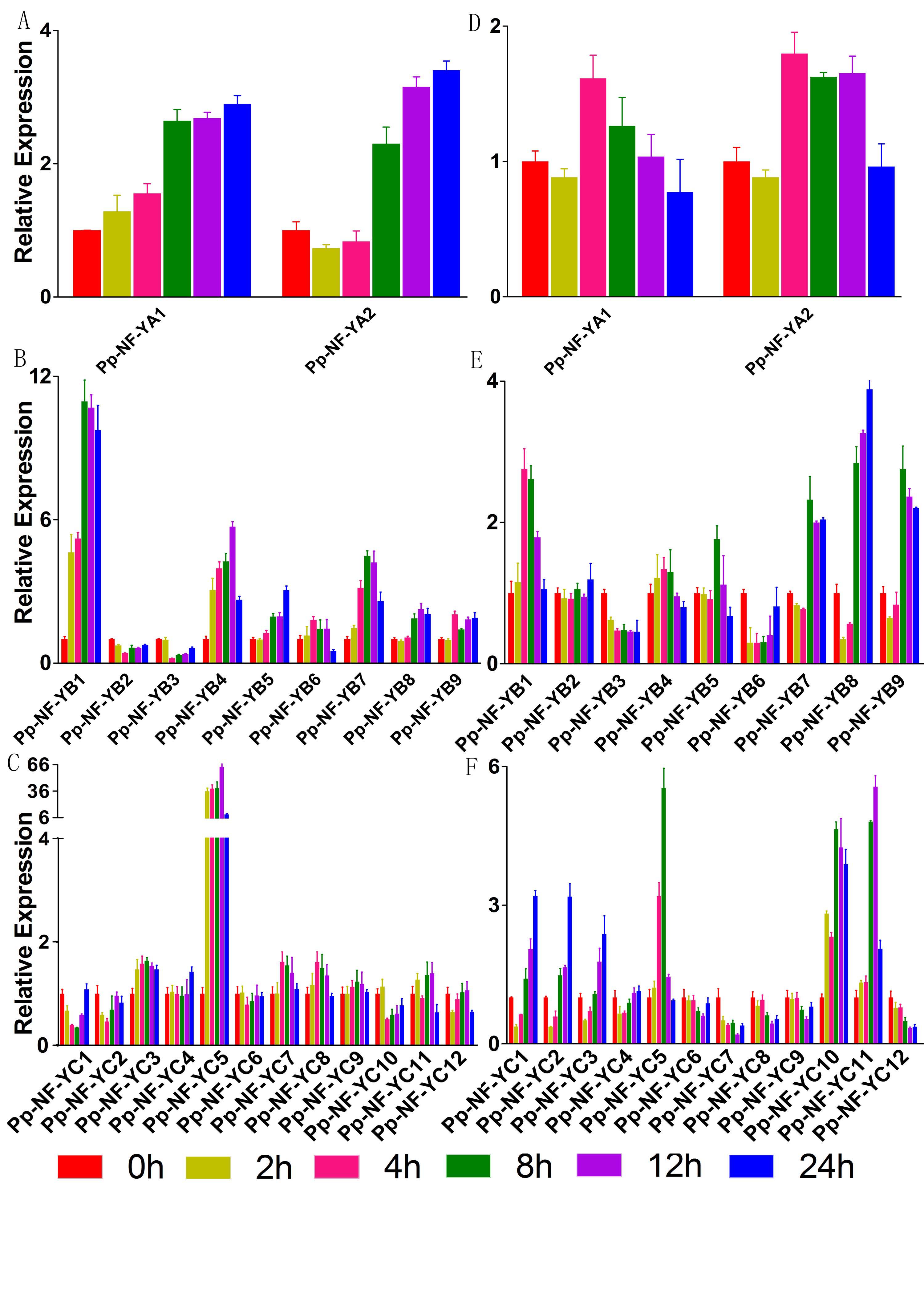

Supplement: Supplementary file 6 [file Image5.JPEG]

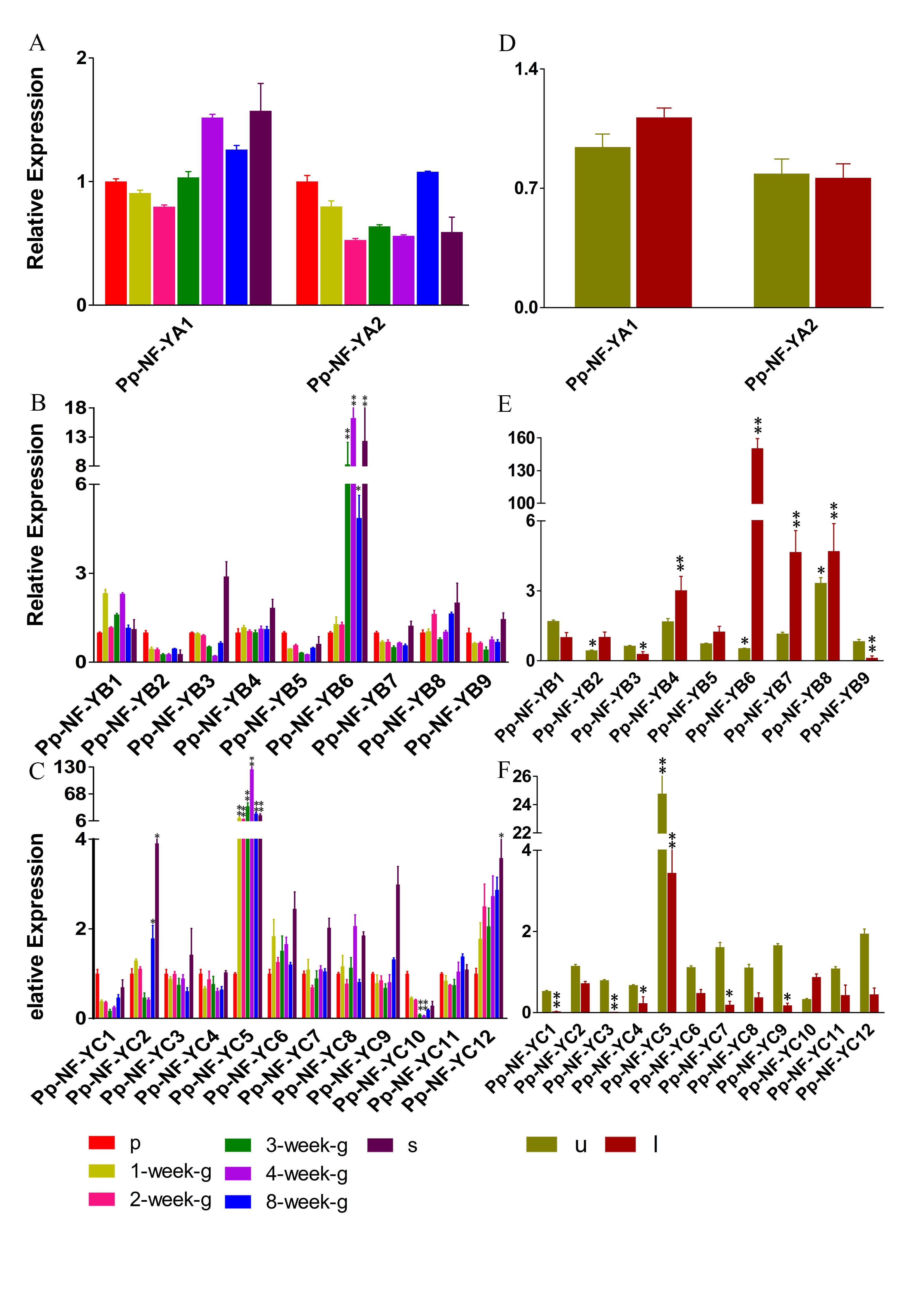

Supplement: Supplementary file 7 [file Image6.JPEG]

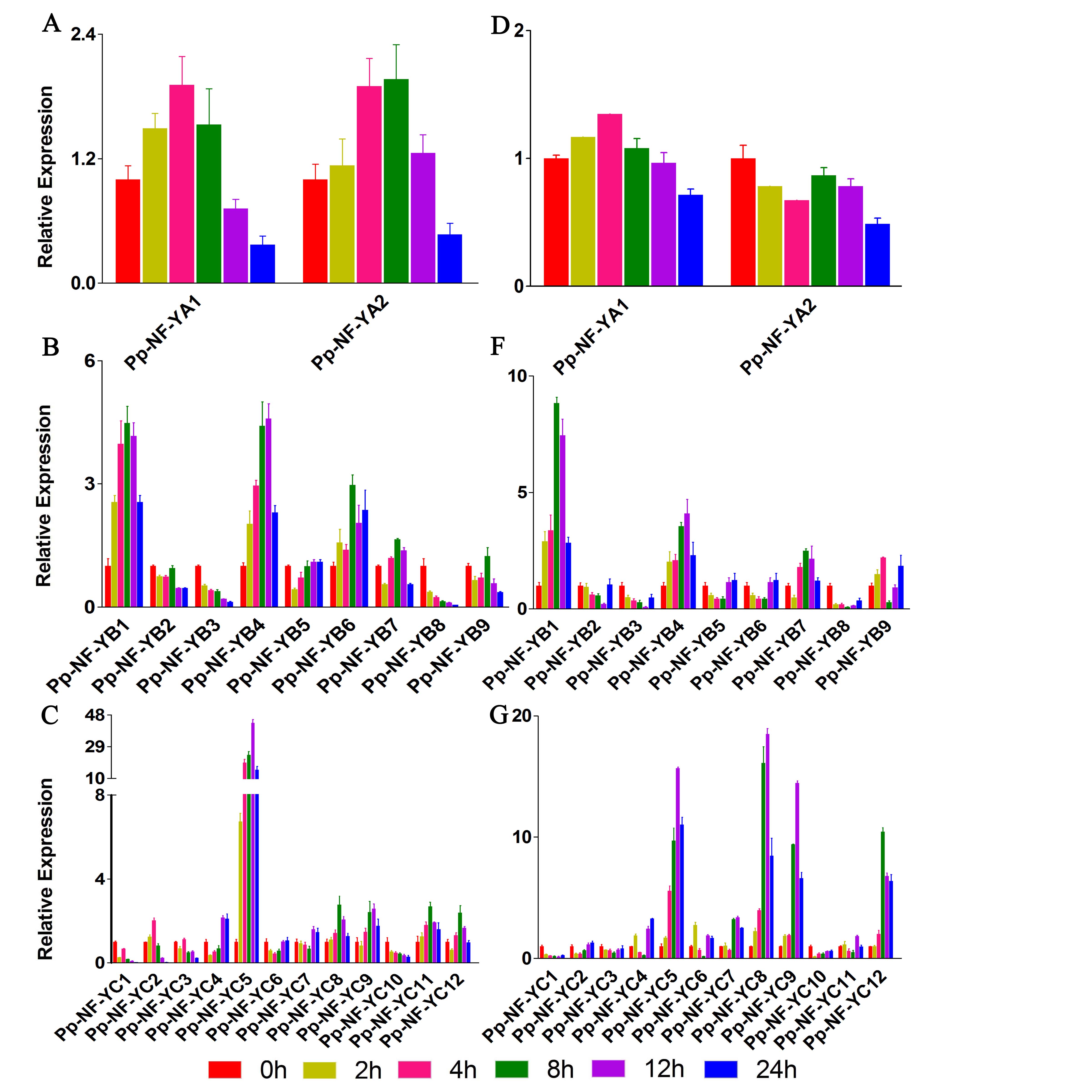

Supplement: Supplementary file 8 [file Image7.JPEG]

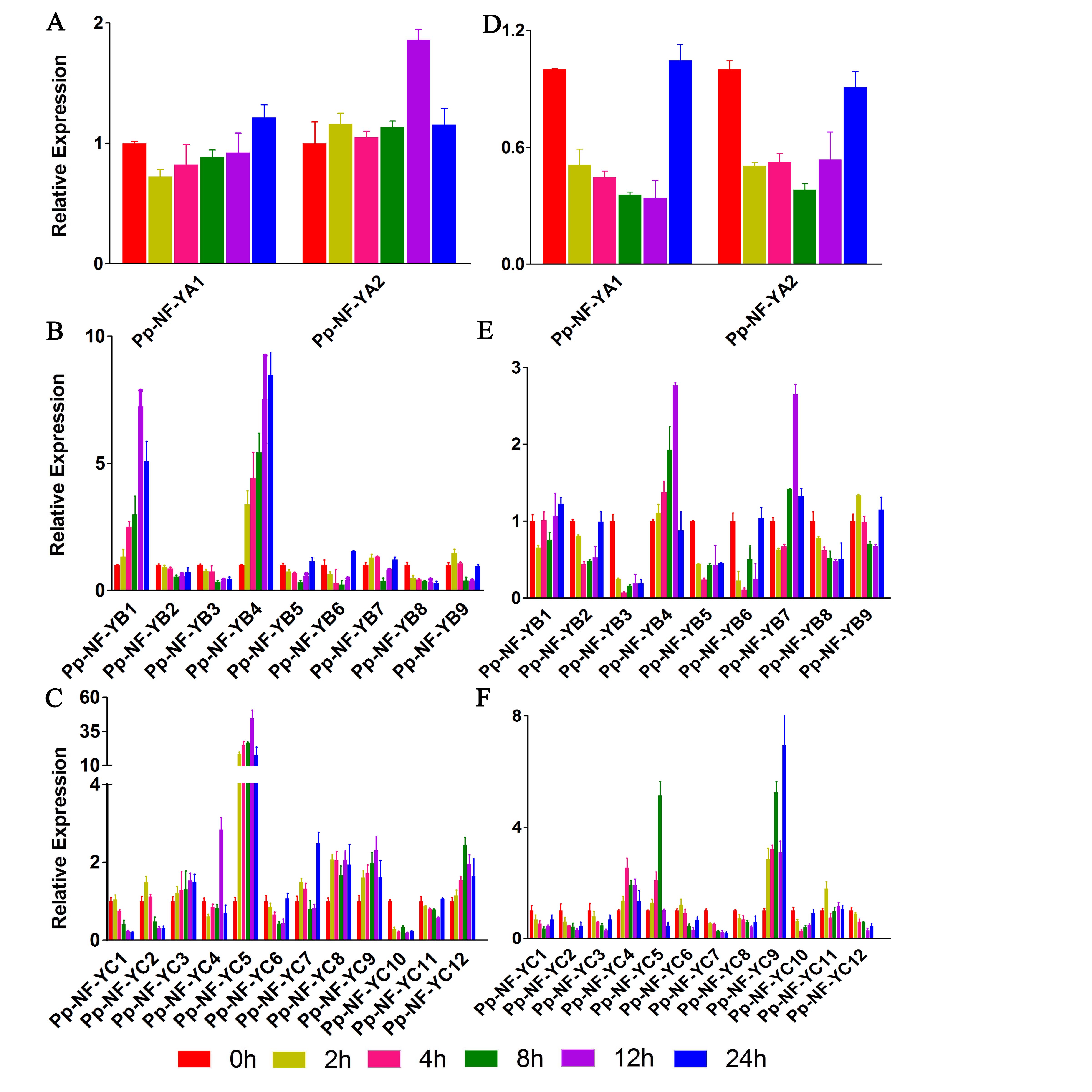

Supplement: Supplementary file 9 [file Image8.JPEG]

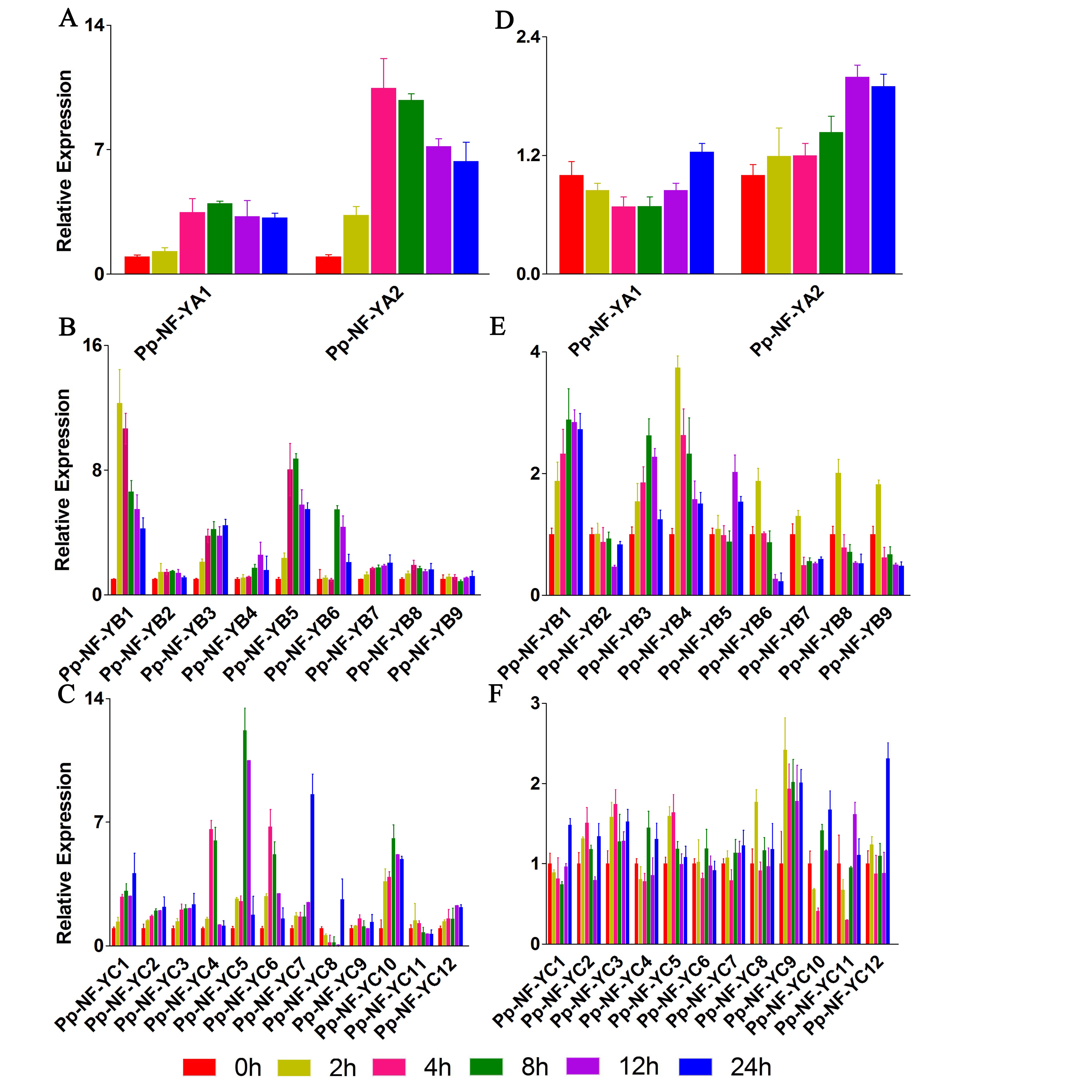

Supplement: Supplementary file 10 [file Image9.JPEG]

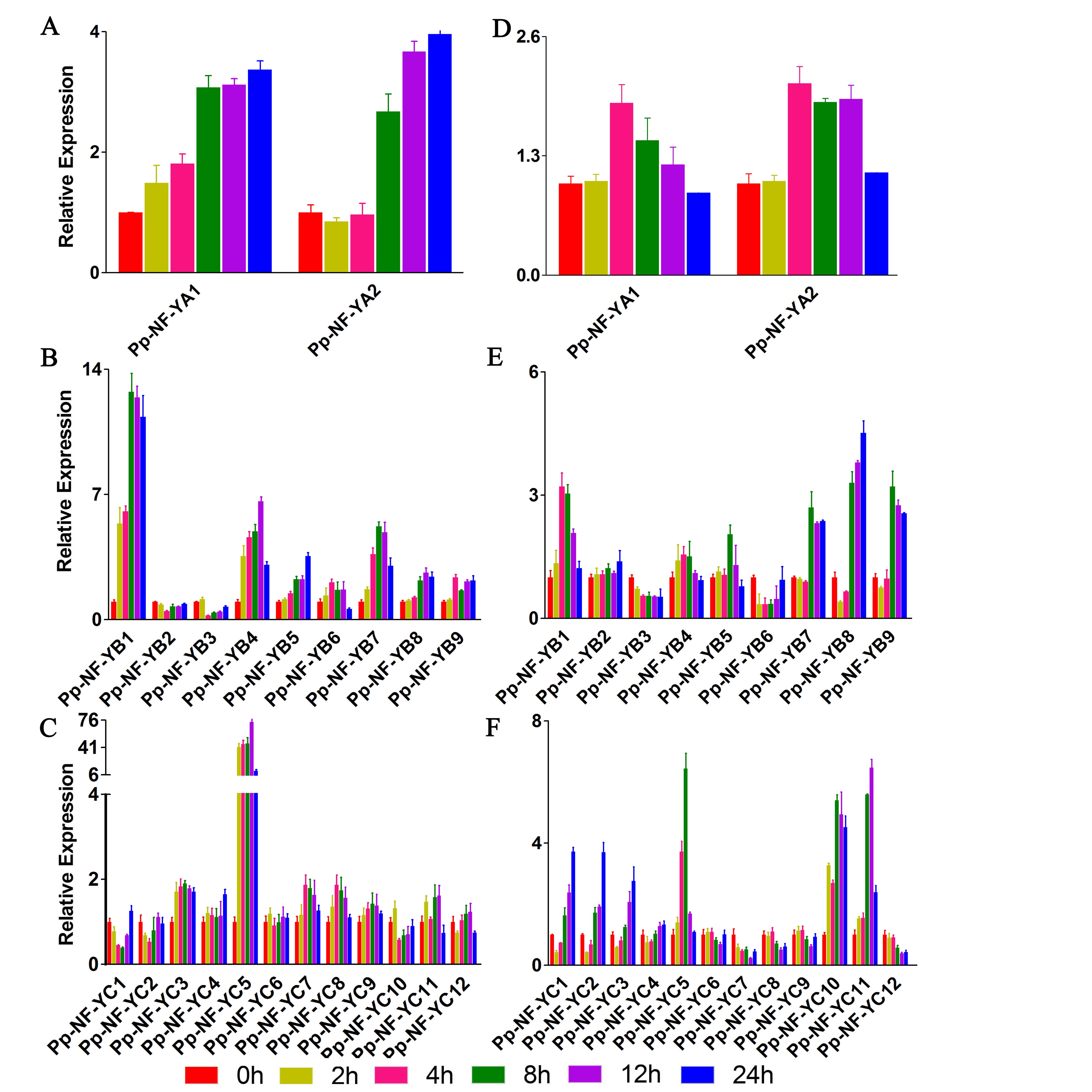

Supplement: Supplementary file 11 [file Image10.JPEG]
